# Stratified glucocorticoid monotherapy is safe and effective for most cases of giant cell arteritis

**DOI:** 10.1093/rap/rkaa024

**Published:** 2020-06-12

**Authors:** Maira Karabayas, Paula Dospinescu, Marc Locherty, Paul Moulindu, Manvi Sobti, Rosemary Hollick, Cosimo De Bari, Susan Robinson, John Olson, Neil Basu

**Affiliations:** r1 Aberdeen Centre for Arthritis & Musculoskeletal Health, University of Aberdeen; r2 Department of Rheumatology; r3 Department of Ophthalmology; r4 Department of Pathology, NHS Grampian, Aberdeen; r5 Institute of Infection, Immunity & Inflammation, University of Glasgow, Glasgow, UK

**Keywords:** giant cell arteritis, temporal artery biopsy, retrospective cohort, glucocorticoid, tapering regimens, visual loss, remission, relapse

## Abstract

**Objectives:**

High-dose glucocorticoids anchor standard care in GCA but are associated with significant toxicity. We aimed to evaluate the safety and effectiveness of a stratified approach to glucocorticoid tapering. The strategy aggressively reduced glucocorticoid doses in those manifesting an adequate early response to treatment, with a view to minimizing glucocorticoid complications.

**Methods:**

A retrospective, population-based study of GCA was performed. All cases were confirmed by temporal artery biopsy between November 2010 and November 2015. Baseline and outcome data were extracted from secondary and primary care records at diagnosis and 1 year follow-up. The primary outcome was loss of vision. Secondary outcomes included remission and relapse rates and CS-related complications.

**Results:**

The cohort consisted of 73 patients (76% female; mean age 73.5 years, s.d. 7.6 years). At presentation, a reduction in visual acuity was recorded in 17 patients (22.3%). The median CRP at diagnosis was 69.5 mg/l [interquartile range (IQR) 40.5–101 mg/l], with a median ESR of 80 mm/h (IQR 60–91 mm/h). At 1 year, remission was achieved in 64 patients (87.7%), whereas 10 patients (13.7%) relapsed. A single patient sustained visual loss after initiation of therapy. The median CRP at 1 year was 4 mg/l (IQR 4–9.5 mg/l) and the mean prednisolone dose was 5.4 mg (0–15 mg). CS-related complications were observed in 10 patients (13.7%).

**Conclusion:**

A stratified approach to CS tapering appeared safe and effective in GCA. It was associated with a high rate of remission and promisingly low rates of relapse at 1 year follow-up. These real-world data indicate that glucocorticoid exposure can be minimized safely in some patients with GCA.

**Key messages**

- A stratified approach to glucocorticoid tapering is effective for most cases of GCA.
- Glucocorticoid exposure can be aggressively, yet safely minimized in selected patients with GCA.

## Introduction

GCA is the commonest form of vasculitis in Caucasians. It has a predilection for the cranial arteries, with patients classically presenting with temporal headache, a spectrum of visual disturbance, jaw claudication and a degree of constitutional upset [1].

The most catastrophic complication of the disease is loss of vision related to myo-intimal proliferation and subsequent vessel occlusion. This is typically unilateral but can be observed bilaterally and is often permanent. In historical cohorts, before the introduction of glucocorticoid (GC) therapy, visual complications were estimated in 35–60% of patients [1]. However, more recent studies suggest that visual loss occurs in 13.2–19.1% of GC-treated patients [2–4]. Although the risk of visual loss reduces significantly once CS therapy is initiated, in clinical practice the fear of inadequate therapy in relationship to this complication might encourage excessive CS use [5].

Historically, GCs have been the mainstay of treatment. A recent review identified a number of CS regimens, although these have not yet been validated, and variability in CS protocols in clinical practice remains an issue [6]. Moreover, GC toxicity is an increasing concern, with data attributing a significant proportion of morbidity to their adverse effects [7]. A GCA cohort analysis showed a significant risk of adverse rates for every 1 g increase in the cumulative GC dose (odds ratio 1.17, 95% CI: 1.06, 1.29) [8].

Research initiatives are directed towards management strategies to reduce the overall burden of GCs, without enhancing the risk of visual compromise [9]. Evidence now supports an effective GC-sparing role for modern biologics, although high financial cost limits their accessibility [10–12]. In other forms of vasculitis, there are now data indicating that historical management protocols incorporate dispensable doses of GC. Aggressive tapering regimens appear to be equivalently effective but with preferable safety profiles. Similar evidence is lacking for GCA and was advocated as a research priority in the 2009 EULAR large vessel vasculitis management recommendations [9].

**Fig. 1:**
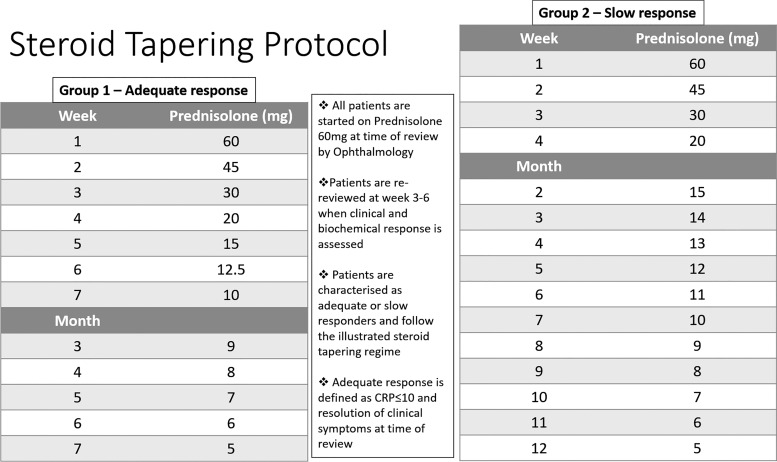
CS tapering protocol

Since 2010, our centre has taken an aggressive approach to GC tapering, with the aim of minimizing treatment-related complications. However, in order to minimize the risk of visual compromise, patients slow to respond are stratified to receive a classical tapering regimen (Fig. 1). We have now conducted a retrospective study to examine the performance of this new GC strategy.

## Methods

We conducted a retrospective observational study on a population-based cohort of GCA in the North East of Scotland. Patients >50 years of age with a positive temporal artery biopsy, as reported by the local pathology department, were included. No ethical approvals were required. The project was registered with the local Research and Development department. 

All temporal artery biopsies carried out between November 2010 and November 2015 in the Grampian region of Scotland (population ∼550 000) were identified from a centralized pathology database. Electronic primary and secondary care records were interrogated to extract patient demographics and disease characteristics at the time of diagnosis and outcomes at 1 year follow-up. Individual primary care practices were contacted to determine an accurate prednisolone dose at 1 year for each patient. Patient demographics included age, sex and smoking status. Baseline disease characteristics assessed included headache, visual disturbance, jaw claudication, constitutional upset and inflammatory markers at diagnosis. The primary outcome examined was loss of vision (defined as complete monocular visual loss) at 1 year. Secondary outcomes included the rate of remission (defined as prednisolone dose ≤7.5 mg and complete resolution of symptoms), rate of relapse (defined as recurrence of symptoms requiring escalation of any immunosuppression) and CS-related complications at 1 year.

In our region, all patients with suspected GCA are immediately started on prednisolone 60 mg in primary care and then fast-tracked to a central neuro-ophthalmology clinic. They are reviewed and clinically assessed within 2 days. Temporal artery biopsy is undertaken at most within 2 weeks of diagnosis. All patients then follow the stratified CS taper illustrated in Fig. 1. Patients are re-assessed between weeks 3 and 6 to determine adequate response (defined as complete resolution of symptoms and CRP <10 mg/l). When clinically appropriate, patients are discharged from the specialist clinic back to primary care with the recommended CS taper as outlined above. All patients remain on 5 mg until the anniversary of diagnosis. They reduce further by 1 mg monthly until CS cessation. In the event of any clinical concerns within primary care, patients are usually re-referred to the neuro-ophthalmology clinic.

The study was a clinical service evaluation and complied with local institutional governance.

## Results

During the 5 year study period, a total of 329 temporal artery biopsies were performed. Of those, 246 were non-diagnostic and 83 were positive for GCA. Of the 83 positive cases, complete outcome data were available for 73.

Our final cohort consisted of 73 patients, 76% of whom were female, with a mean age at diagnosis of 73.5 (s.d. 7.6) years. At the time of diagnosis, objective monocular reduction in visual acuity relating to GCA was recorded in 17 patients (22.3%). More specifically, 11 patients presented with anterior ischaemic optic neuropathy, 3 with central retinal artery occlusion, 2 with sixth nerve palsy and 1 with posterior ischaemic optic neuropathy. However, 39 patients (53.4%) reported subjective visual disturbances ranging from undifferentiated visual impairment (*n* = 20) to blurry vision (*n* = 10) and diplopia (*n* = 8). Jaw claudication was reported in 34 patients (46.6%) in the cohort, and 37 (50.7%) described a degree of constitutional upset. On examination, 20 (38.4%) reported temporal artery tenderness. All patients fulfilled ACR criteria 1990 [13]. At the time of diagnosis, the median CRP was 69.5 mg/l [interquartile range (IQR) 40.5–101 mg/l] and the median ESR was 80 mm/h (IQR 60–91 mm/h) (Table 1).

**Table 1:** Patient demographics and baseline disease characteristics

Characteristic	Value
Age, mean (s.d.), years	73.5 (7.6)
Female, *n* (%)	56 (76)
ACR criteria 1990 fulfilled, *n* (%)	73 (100)
Headache, *n* (%)	65 (89)
Jaw claudication, *n* (%)	34 (46.6)
Constitutional upset, *n* (%)	37 (50.7)
Subjective visual impairment, *n* (%)	39 (53.4)
Objective reduction in visual acuity, *n* (%)	17 (22.3)
Temporal artery tenderness, *n* (%)	20 (38.4)
CRP, median (interquartile range), mg/l	69.5 (40.5–101)
ESR, median (interquartile range), mm/h	80 (60–91)

At 1 year, remission was achieved in 64 patients (87.7%) of the cohort, whereas 10 (13.7%) relapsed. The reported relapses were mainly in the form of headache and constitutional upset requiring temporary escalation in prednisolone dose. A single patient sustained visual loss from initiation of therapy. The median CRP at 1 year was 4 mg/l (IQR 4–9.5 mg/l), with a mean prednisolone dose of 5.4 mg (0–15 mg), and two patients required additional immunosuppression. The mean discharge time from neuro-ophthalmology clinic was 18.3 weeks (s.d. 17 weeks). CS-related complications were observed in 15 patients (20.6%), cataract in 4, recurrent urinary tract infections *n* = 3, thoracic wedge fracture *n* = 2, avascular necrosis of femoral head *n* = 1, pelvic fracture in 1, CS-induced diabetes in 1, CS-induced psychosis in 1, intra-abdominal sepsis requiring hospital admission in 1 and osteoporosis in 1. Finally, the estimated cumulative prednisolone dose for the aggressive CS taper (group 1) was 2997.5 mg, and the estimated cumulative dose for the slower group was 4385mg. It was not possible to evaluate differences in outcomes between CS regimens.

## Discussion

In this single-centre observational retrospective study of a service which adopts an aggressive GC minimization taper in early GC responders, visual loss was rare (1.4%), despite patients tapering to 20 mg of prednisolone by 4 weeks. The majority of patients achieved disease remission (87.7%), and relapse was uncommon (13.7%).

Our low observed rates of visual loss align with those reported from another population-based cohort. Salvarani *et al.* [14] examined visual manifestations in Italian patients with biopsy-proven GCA (*n* = 136). They reported a single patient sustaining permanent visual loss 14 months from the initiation of treatment. Aiello *et al.*[2] found a 1% 5 year probability of developing new visual loss from initiation of therapy in an era where GC dosing was significantly higher.

Disease remission was achieved by 87.7% of the cohort. In general, existing literature indicates that only 15–20% of patients achieve this disease state with GC monotherapy [10]. Furthermore, our promisingly low rates of relapse (13.7%) also appear favourable in comparison to the extant literature, where typically rates between 34 and 74.5% have been reported [15, 16]. There are a few reasons for these discrepancies. First, remission and relapse rates vary significantly within clinical trials and population-based cohort studies. These figures should be interpreted cautiously given the difficulty of adjusting for confounding factors such as disease duration, cumulative CS use and follow-up time. Second, there are significant variations in the definitions used for remission and relapse, which have only been standardized recently in the updated EULAR recommendations [12]. It is hoped that future studies will assess these newly homogenized outcomes in a more systematic manner to inform clinical practice. Third, our cohort consisted of patients presenting with cranial symptoms. It has been shown that cranial GCA is usually associated with a more monophasic disease course [17]. That said, we did not routinely perform specialist imaging to confirm the presence or absence of extracranial artery involvement. Fourth, the majority of the literature is sourced from specialized centre sampling frames. More recent population-level real-world data provide evidence for low relapse rates [18]. Although these data align with our observations, it should be noted that this was a conference abstract publication, with inherent limitations and potential methodical flaws and has not been peer reviewed. Finally, we speculate that our fast-track service has enabled very early diagnosis, which allowed for the application of a stratified approach to GC tapering, in turn conferring favourable long-term outcomes. Prognostic benefits of early therapy are certainly now established across other rheumatic disorders [19].

Adverse events relating to GC therapy at 1 year were prevalent. The estimated cumulative prednisolone dose for the aggressive CS taper (group 1) was 2997.5 mg, a value which compares directly with the 26 week CS placebo group (3296 mg) and significantly less than the 56 week steroid placebo group, in the GiACTA trial (3818 mg) and the group 2 CS taper (4385 mg), which mirrors classical regimens [11]. It is important to highlight that the values related to our cohort assume full compliance with the taper protocol. Owing to difficulties with data capturing, we were not able to confirm this retrospectively for each individual patient to provide an accurate mean cumulative dose.

Several other limitations should also be considered. Firstly, this study is retrospective in design and dependent on electronic records for data extraction. In our region, however, electronic records are centralized, including laboratory, clinical and pathology records. In addition, a direct link between primary and secondary care made it feasible to capture a wide range of data. Incomplete data capture remained a challenge; for example, it was not possible to characterize group assignment confidently. The mean discharge time from the ophthalmology clinic to primary care was 18.3 weeks (s.d. 17 weeks) from the time of diagnosis. Adherence to the standardized CS taper was assumed in the absence of any referrals back to ophthalmology or rheumatology clinics or any relapses documented in primary care records. To account for that, we also contacted individual practices to obtain an accurate personalized prednisolone dose at 1 year for all patients. Secondly, outcomes were examined only at 1 year, and it is possible that we might have failed to capture later relapses. A longitudinal study cohort showed a mean time to first relapse of 79 (s.d. 75) weeks [range 11–339 weeks, median 51 (IQR, 89) weeks], but 50% of these patients relapsed within the first year [20].

Thirdly, in order to ensure that our cohort consisted of patients with a true diagnosis of GCA, we elected to include only those with a positive temporal artery biopsy. Owing to the lack of sensitivity of temporal artery biopsy, we might have missed cases. Our patient demographics align with epidemiological predictions predominantly affecting women (3.3:1), with a mean age at diagnosis of 73.5 (s.d. 7.6) years [21]. A retrospective analysis examining the incidence of GCA in the UK between 1990 and 2001 suggested that the age-standardized incidence ratio of GCA in Scotland is 67 (95% CI: 54, 82), a figure which directly compares to our cohort [22]. Taken together, we are likely to have captured a representative cohort. What transpires from this retrospective study is that having a centralized, fast-track service allows the application of an aggressive, stratified CS taper to reduce cumulative CS burden, whilst ensuring that patient outcomes are optimal.

Although our study demonstrates reassuring outcomes, these findings are preliminary. Adoption of this stratified CS taper in different populations would be required to validate these observations further and correct for some of the limitations discussed. A larger prospective longitudinal study is desirable to quantify GC exposure more precisely and to characterize further the morbidity relating to both disease and treatment. This would make it possible to differentiate and phenotypically characterize the two CS taper groups, in an attempt to inform our future practice as the field finally moves towards to an era of personalized medicine.

## Conclusions

In conclusion, in this retrospective observational study of a real-world population cohort in the North East of Scotland, a stratified approach to corticosteroid therapy, which leveraged initial treatment response as a method to triage patients towards aggressive steroid tapering, appears to be an effective model of the GCA pathway. It was associated with high rates of remission and promisingly low rates of relapse at 1 year follow-up. These real-world data suggest that glucocorticoid exposure could safely be reduced compared with classical regimens when integrated in fast-track pathways with early specialist input.

*Funding*: We are grateful to Versus Arthritis (grant 12159) for supporting our work.

*Disclosure statement:* N.B. has received non-promotional speaking fees from Roche, Abbvie, Vifor, Lilly and Pfizer and research funding from Pfizer, GSK, Vifor and Novartis. The other authors have declared no conflicts of interest.
